# Supplementary material for: Synthesis of 2,2,6-Trisubstituted 5-Methylidene-tetrahydropyran-4-ones with Anticancer Activity
Source: Molecules. 2020 Jan 30;25(3):611. doi: 10.3390/molecules25030611 (PMC7038078; doi:10.3390/molecules25030611)
Supplement: Supplementary file 1 [file molecules-25-00611-s001.zip › Supporting information.docx]

**Synthesis of 2,2,6-trisubstituted 5-methylidene- tetrahydropyran-4-ones with anticancer activity**

Tomasz Bartosik^1^, Jacek Kędzia^1^ Joanna Drogosz-Stachowicz^2^, Anna Janecka^2,^, Urszula Krajewska^3^, Marek Mirowski^3^ and Tomasz Janecki^1,^*

^1^ Institute of Organic Chemistry, Lodz University of Technology, Żeromskiego 116, 90-924 Łódź, Poland; [tomasz.bartosik@p.lodz.pl](mailto:tomasz.bartosik@p.lodz.pl) (T.B), [jacek.kedzia@p.lodz.pl](mailto:jacek.kedzia@p.lodz.pl) (J.K)

^2^ Department of Biomolecular Chemistry, Medical University of Łódź, Mazowiecka 6/8, 92-215 Łódź, Poland; [anna.janecka@umed.lodz.pl](mailto:anna.janecka@umed.lodz.pl) (A.J.); [joanna.drogosz@studumed.lodz.pl](mailto:joanna.drogosz@studumed.lodz.pl) (J.D.)

^3^ Department of Pharmaceutical Biochemistry and Molecular Diagnostics, Faculty of Pharmacy, Medical University of Łódź, Muszyńskiego 1, 90-151, Łódź, Poland; [marek.mirowski@umed.lodz.pl](mailto:marek.mirowski@umed.lodz.pl) (MM); ukrajewska@o2.pl (UK)

***** Correspondence: [tomasz.janecki@p.lodz.pl](mailto:tomasz.janecki@p.lodz.pl) (T.J); Tel.: +48 426313220

**Supporting Information**

Table of Contents

1. General information. 2

2. General procedures and characterization data. 2

2.1. Synthesis of diethyl (4-hydroxy-2-oxo-4,4-dialkyl(aryl)butyl)phosphonates (**7a – d**) 2

2.2. Synthesis of diethyl (2,2-dialkyl(diaryl)-4-oxo-3,4-dihydro-2*H*-pyran-5-yl)

phosphonates (**9a – c**) 3

2.3. Synthesis of diethyl (4'-oxo-3',4'-dihydrospiro[fluorene-9,2'-pyran]-5'-yl)phosphonate (**9d**) 4

2.4. Synthesis of diethyl (2-alkyl-6,6-alkyl(aryl)-4-oxotetrahydro-2*H*-pyran-3-yl)phosphonates

(**10a-c**, **10e-g**, **10i-k**, **10m-o**) 4

2.5. Synthesis of diethyl (2-aryl-6,6-alkyl(aryl)-4-oxotetrahydro-2*H*-pyran-3-yl)phosphonates

(**10d**, **h, l** and **p**) 9

3. Copies of ^31^P, ^1^H and ^13^C NMR Spectra. 12

1. General information.

NMR spectra were recorded on a Bruker DPX 250 or Bruker Avance II instrument at 250.13 MHz or 700 MHz for ^1^H, 62.9 MHz or 176 MHz for ^13^C, and 101.3 MHz or 283 MHz for ^31^P NMR using tetramethylsilane as internal and 85% H_3_PO_4_ as external standard. ^31^P NMR spectra were recorded using broadband proton decoupling. IR spectra were recorded on a Bruker Alpha ATR spectrophotometer. Melting points were determined in open capillaries and are uncorrected. Column chromatography was performed on Aldrich® silica gel 60 (230–400 mesh). Thin-layer chromatography was performed with precoated TLC sheets of silica gel 60 F254 (Aldrich®). The purity of tested compounds was determined by combustion elemental analyses (CHN, elemental analyzer EuroVector 3018, Elementar Analysen systeme GmbH). MS spectra of intermediates were performed on combined Waters 2695-Waters ZQ 2000 LC/MS apparatus. EI mass spectra of final compounds were recorded on a GCMS-QP2010 ULTR A instrument (Shimadzu, Kioto, Japan). The mass spectra were obtained using the following operating conditions: electron energy of 70 eV and ion source temperature of 200°C. Samples were introduced via a direct insertion probe heated from 30 to 300°C. Reagents and starting materials were purchased from commercial vendors and used without further purification. All organic solvents were dried over appropriate drying agents and distilled prior to use. Standard syringe techniques were used for transferring dry solvents. All flasks are flame drying and flushed with argon.

2. General procedures and characterization data.

2.1 Synthesis of diethyl (4-hydroxy-2-oxo-4,4-dialkyl(aryl)butyl)phosphonates (**7a–d**)

In a round-bottomed three – necked flask under argon atmosphere NaH (0.51 g, 17.00 mmol, 80% in mineral oil) was suspended in THF (48 mL). The suspension was stirred and solution of diethyl 2-oxopropylphosphonate (3.00 g, 15,45 mmol) in THF (3 mL) was added dropwise. The reaction mixture was stirred for 30 min, cooled below – 30 ⁰C in dry ice – acetone bath and n-butyllithium (6.80 mL of 2.5 M solution in hexanes, 17.00 mmol) was added dropwise. Reaction mixture was stirred for 30 minutes at this temperature, cooled to -78 ⁰C in dry ice – acetone bath and solution of ketone (18.54 mmol) in THF (6 mL) was added dropwise. Reaction mixture was stirred for 1.5 h at this temperature. After this time the reaction was quenched by adding saturated solution of ammonium chloride (100 mL). The water layer was extracted with DCM (3 × 50 mL), the organic layers were combined, washed with brine and dried over MgSO_4_. The solvents were evaporated under reduced pressure and the resulting crude product was purified by column chromatography (eluent ethyl acetate).

Diethyl (4-hydroxy-4-methyl-2-oxopentyl)phosphonate (**7a**) (2.58 g, 66%) Colorless oil. ^31^P NMR (283 MHz, Chloroform-*d*) δ 19.65. ^1^H NMR (700 MHz, Chloroform-*d*) δ 1.23 (s, 6H), 1.31 (t, *J* = 7.1 Hz, 6H), 2.78 (s, 2H), 3.09 (d, *J* = 22.9 Hz, 2H), 3.64 (s, 1H), 4.02 – 4.19 (m, 4H). ^13^C NMR (176 MHz, Chloroform-*d*) δ 16.38 (d, *J* = 6.3 Hz, 2$\times$C), 29.38 (2$\times$C), 43.89 (d, *J* = 126.5 Hz), 55.12, 62.79 (d, *J* = 6.5 Hz, 2$\times$C), 69.76, 203.64 (d, *J* = 6.2 Hz). ESI-MS [M+Na]^+^ = 275.1. Anal. Calcd for C_10_H_21_O_5_P: C, 47.62; H, 8.39. Found: C, 47.51; H, 8.37.

Diethyl (3-(1-hydroxycyclohexyl)-2-oxopropyl)phosphonate (**7b**) (3.75 g, 83%) Colorless oil. ^31^P NMR (101 MHz, Chloroform-*d*) δ 20.12 . ^1^H NMR (700 MHz, Chloroform-*d*) δ 1.18 – 1.26 (m, 1H), 1.27 – 1.31 (m, 6H) 1.33 – 1.42 (m, 4H), 1.43 – 1.51 (m, 1H), 1.53 – 1.68 (m, 4H), 2.74 (s, 2H), 3.09 (d, *J* = 22.9 Hz, 2H), 3.46 (s, 1H), 4.02 – 4.17 (m, 4H). ^13^C NMR (176 MHz, Chloroform-*d*) δ 16.36 (d, *J* = 6.3 Hz, 2$\times$C), 21.94 (2$\times$C), 25.66, 37.60(2$\times$C), 44.07 (d, *J* = 126.4 Hz), 54.24, 62.71 (d, *J* = 6.5 Hz, 2$\times$C), 70.86 , 203.66 (d, *J* = 5.9 Hz). ESI-MS [M+Na]^+^ = 315.1. Anal. Calcd for C_13_H_25_O_5_P: C, 53.42; H, 8.62. Found: C, 53.33; H, 8.61.

Diethyl (4-hydroxy-2-oxo-4,4-diphenylbutyl)phosphonate (**7c**) (4.84 g, 83%) White crystal, mp 59-60 °C.  ^31^P NMR (284 MHz, Chloroform-*d*) δ 18.98. ^1^H NMR (700 MHz, Chloroform-*d*) δ 1.29 (t, *J* = 7.1 Hz, 6H), 3.06 (d, *J* = 23.1 Hz, 2H), 3.66 (s, 2H), 3.99 – 4.13 (m, 4H), 4.77 (s, 1H), 7.17 – 7.21 (m, 2H), 7.26 – 7.30 (m, 4H), 7.41 – 7.45 (m, 4H). ^13^C NMR (176 MHz, Chloroform-*d*) δ 16.34 (d, *J* = 5.9 Hz, 2$\times$C), 43.84 (d, *J* = 126.0 Hz), 54.27, 62.86 (d, *J* = 6.4 Hz, 2$\times$C), 76.92, 125.70 (4$\times$C), 127.06 (2$\times$C), 128.30 (4$\times$C), 146.11 (2$\times$C), 202.95 (d, *J* = 5.7 Hz). ESI-MS [M+Na]^+^ = 399.1. Anal. Calcd for C_20_H_25_O_5_P: C, 63.82; H, 6.70. Found: C, 63.90; H, 6.68.

Diethyl (3-(9-hydroxy-9H-fluoren-9-yl)-2-oxopropyl)phosphonate (**7d**) (4.45 g, 77%) Yellow crystal, mp 68-69 °C. ^31^P NMR (284 MHz, Chloroform-*d*) δ 19.02 . ^1^H NMR (700 MHz, Chloroform-*d*) δ 1.20 (t, *J* = 7.1 Hz, 6H), 3.01 (d, *J* = 22.8 Hz, 2H), 3.19 (s, 2H), 3.92 – 4.01 (m, 4H), 4.51 (s, 1H), 7.23 – 7.26 (m, 2H), 7.29 – 7.35 (m, 2H), 7.47 – 7.52 (m, 2H), 7.56 – 7.60 (m, 2H). ^13^C NMR (176 MHz, Chloroform-*d*) δ 16.22 (d, *J* = 6.3 Hz, 2$\times$C), 43.73 (d, *J* = 126.5 Hz), 52.35 , 62.63 (d, *J* = 6.6 Hz, 2$\times$C), 80.26 , 120.06 (2$\times$C), 124.00 (2$\times$C), 128.00 (2$\times$C), 129.18 (2$\times$C), 139.19 (2$\times$C), 147.86 (2$\times$C), 202.36 (d, *J* = 6.4 Hz). ESI-MS [M+Na]^+^ = 397.1. Anal. Calcd for C_20_H_23_O_5_P: C, 64.17; H, 6.19. Found: C, 64.02; H, 6.21.

2.2 Synthesis of diethyl (2,2-dialkyl(diaryl)-4-oxo-3,4-dihydro-2*H*-pyran-5-yl)phosphonate (**9a–c**)

The solution of diethyl (4-hydroxy-2-oxo-4,4-dialkyl(aryl)butyl)phosphonate (**7a–c**) (10.00 mmol), in dry toluene (100 mL) was warmed to 100 ⁰C. After 15 minutes dimethyl formamide dimethyl acetal (DMF – DMA) (30.00 mmol, 4.24 mL) was added dropwise and stirred for 45 minutes. After this time to reaction mixture boron trifluoride etherate (BF_3_ $\cdot$ Et_2_O) (10.00 mmol, 1.24 mL) was added dropwise. Reaction was controlled by ^31^P NMR. After completion of the reaction, the reaction mixture was cooled to room temperature and the solution of saturated sodium hydrocarbonate (100 mL) was added. The water layer was extracted with ethyl acetate (3 × 50 mL), the organic layers were combined, washed with brine and dried over MgSO_4_. The solvents were evaporated under reduced pressure and the resulting crude product was purified by column chromatography (eluent ethyl acetate).

Diethyl (2,2-dimethyl-4-oxo-3,4-dihydro-2*H*-pyran-5-yl)phosphonate (**9a**) (2.2 g, 84%) Colourless oil. ^31^P NMR (101 MHz, Chloroform-*d*) δ 14.93. ^1^H NMR (700 MHz, Chloroform-*d*) δ 1.25 (t, *J* = 7.1 Hz, 6H), 1.40 (s, 6H), 2.50 (d, *J* = 1.3 Hz, 2H), 3.86 – 4.21 (m, 4H), 7.92 (d, *J* = 9.5 Hz, 1H). ^13^C NMR (176 MHz, Chloroform-*d*) δ 16.32 (d, *J* = 6.4 Hz, 2$\times$C), 26.05 (2$\times$C), 47.80 (d, *J* = 7.6 Hz), 62.29 (d, *J* = 4.2 Hz, 2$\times$C), 83.63, 105.68 (d, *J* = 194.0 Hz), 170.80 (d, *J* = 19.8 Hz), 189.04 (d, *J* = 2.4 Hz). ESI-MS [M+Na]^+^ = 285.1. Anal. Calcd for C_11_H_19_O_5_P: C, 50.38; H, 7.30. Found: C, 50.23; H, 7.32.

Diethyl (4-oxo-1-oxaspiro[5.5]undec-2-en-3-yl)phosphonate (**9b**) (2.72 g, 90%) Colourless oil. ^31^P NMR (101 MHz, Chloroform-*d*) δ 15.09. ^1^H NMR (700 MHz, Chloroform-*d*) δ 1.30 (t, *J* = 7.1 Hz, 6H), 1.43 – 1.55 (m, 4H), 1.56 – 1.68 (m, 3H), 1.88 – 2.01 (m, 3H), 2.52 (s, 2H), 4.03 – 4.18 (m, 4H), 8.00 (d, *J* = 9.4 Hz, 1H). ^13^C NMR (176 MHz, Chloroform-*d*) δ 16.42 (d, *J* = 6.5 Hz, 2$\times$C), 21.39 (2$\times$C), 24.93, 34.43 (2$\times$C), 47.10, 62.37 (d, *J* = 4.7 Hz, 2$\times$C), 84.75, 106.04 (d, *J* = 194.4 Hz), 170.62 (d, *J* = 20.1 Hz), 189.16 (d, *J* = 2.6 Hz). ESI-MS [M+Na]^+^ = 325.1. Anal. Calcd for C_14_H_23_O_5_P: C, 55.62; H, 7.67. Found: C, 55.70; H, 7.65.

Diethyl (4-oxo-2,2-diphenyl-3,4-dihydro-2*H*-pyran-5-yl)phosphonate (**9c**) (2.71 g, 70%) Pale yellow oil. ^31^P NMR (101 MHz, Chloroform-*d*) δ 13.89. ^1^H NMR (700 MHz, Chloroform-*d*) δ 1.14 (t, *J* = 7.1 Hz, 6H), 3.32 (d, *J* = 1.2 Hz, 2H), 3.74 – 3.86 (m, 2H), 3.87 – 3.99 (m, 2H), 7.27 – 7.38 (m, 10H), 8.14 (d, *J* = 9.4 Hz, 1H). ^13^C NMR (176 MHz, Chloroform-*d*) δ 16.06 (d, *J* = 6.5 Hz, 2$\times$C), 47.62 (d, *J* = 7.6 Hz), 62.23 (d, *J* = 5.5 Hz, 2$\times$C), 89.63, 108.14 (d, *J* = 190.1 Hz), 126.14 (4$\times$C), 128.51 (2$\times$C), 128.62 (4$\times$C), 141.01 (2$\times$C), 170.93 (d, *J* = 20.0 Hz), 188.12 (d, *J* = 2.0 Hz). ESI-MS [M+Na]^+^ = 409.0. Anal. Calcd for C_21_H_23_O_5_P: C, 65.28; H, 6.00. Found: C, 65.42; H, 6.02.

2.3 Synthesis of diethyl (4'-oxo-3',4'-dihydrospiro[fluorene-9,2'-pyran]-5'-yl)phosphonate (**9d**)

Diethyl (3-(9-hydroxy-9H-fluoren-9-yl)-2-oxopropyl)phosphonate (**7d**) (10.00 mmol) was dissolved in dry toluene (100 mL) at room temperature. After 15 minutes dimethyl formamide dimethyl acetal (DMF – DMA) (30.00 mmol, 4.24 mL) was added dropwise and stirred for 45 minutes. After this time to reaction mixture boron trifluoride etherate (BF_3_ $\cdot$ Et_2_O) (10.00 mmol, 1.24 mL) was added dropwise and stirred overnight. After completion of the reaction the solution of saturated sodium hydrocarbonate (100 mL) was added. The water layer was extracted with ethyl acetate (3 × 50 mL), the organic layers were combined, washed with brine and dried over MgSO_4_. The solvents were evaporated under reduced pressure and the resulting crude product was purified by column chromatography (eluent ethyl acetate).

Diethyl (4'-oxo-3',4'-dihydrospiro[fluorene-9,2'-pyran]-5'-yl)phosphonate (**9d**) (2.54 g, 66%) Yellow crystal, mp 120 - 121 °C.^31^P NMR (101 MHz, Chloroform-*d*) δ 13.98 . ^1^H NMR (700 MHz, Chloroform-*d*) δ 1.40 (t, *J* = 7.1 Hz, 6H), 3.04 (d, *J* = 1.3 Hz, 2H), 4.19 – 4.33 (m, 4H), 7.28 (dd, *J* = 8.6, 7.5 Hz, 2H), 7.43 (dd, *J* = 8.6, 7.5 Hz, 2H), 7.53 (dd, *J* = 8.6, 7.5 Hz, 2H), 7.63 (dd, *J* = 8.6, 7.5 Hz, 2H), 8.24 (d, *J* = 9.7 Hz, 1H). ^13^C NMR (176 MHz, Chloroform-*d*) δ 16.54 (d, *J* = 6.4 Hz, 2$\times$C), 44.12 (d, *J* = 7.6 Hz), 62.67 (d, *J* = 5.8 Hz, 2$\times$C), 89.95, 107.60 (d, *J* = 195.3 Hz), 120.62 (2$\times$C), 124.12 (2$\times$C), 128.69 (2$\times$C), 130.89 (2$\times$C), 139.75 (2$\times$C), 143.52 (2$\times$C), 172.26 (d, *J* = 19.7 Hz), 188.71 (d, *J* = 2.4 Hz). ESI-MS [M+Na]^+^ = 407.0. Anal. Calcd for C_21_H_21_O_5_P: C, 65.62; H, 5.51. Found: C, 65.42; H, 5.52.

2.4 Synthesis of diethyl (2-alkyl-6,6-alkyl(aryl)-4-oxotetrahydro-2H-pyran-3-yl)phosphonates (**10a-c**, **e-g**, **i-k**, **m-o**)

The solution of (2,2-dialkyl(diaryl)-4-oxo-3,4-dihydro-2*H*-pyran-5-yl)phosphonates (**9a–d**) (1.0 mmol) in dry THF (10 mL) was cooled to 0 ⁰C in ice – water bath and Grignard reagent (3.0 mmol in THF) was added dropwise in argon atmosphere. After 2 hours at this temperature, saturated solution of ammonium chloride (15 mL) was added. The water layer was washed with DCM (3 x15 mL). Combined organic extracts were washed with brine (20 mL) and dried over MgSO_4_. The solvents were evaporated under reduced pressure and the resulting crude product was purified by column chromatography (eluent DCM : Acetone 20:1).

Diethyl (2-ethyl-6,6-dimethyl-4-oxotetrahydro-2*H*-pyran-3-yl)phosphonate (**10a**) (204.6 mg, 70%) Colorless oil. ^31^P NMR (101 MHz, Chloroform-*d*) δ 19.07 (trans), 19.44 (cis), 25.93 (enol). ^1^H NMR (700 MHz, Chloroform-*d*) δ 0.97 (t, *J* = 7.3 Hz, 3H trans + 3H cis), 1.15 (s, 3H, cis), 1.20 (s, 3H, trans), 1.28 – 1.34 (m, 9H trans + 6H cis), 1.36 (s, 3H, cis), 1.43 – 1.53 (m, 1H, trans), 1.75 – 1.84 (m, 1H, cis), 1.88 – 1.94 (m, 1H, cis), 1.94 – 2.00 (m, 1H, trans), 2.24 (dd, *J* = 13.5, 1.6 Hz, 1H, cis), 2.29 (dd, *J* = 13.2, 1.2 Hz, 1H, trans), 2.79 – 2.88 (m, 3H, 2H trans + 1H cis), 2.95 (ddd, *J* = 21.3, 3.4, 1.7 Hz, 1H, cis), 3.81 (dddd, *J* = 38.8, 8.5, 5.0, 3.4 Hz, 1H, cis), 4.04 – 4.19 (m, 4H trans + 4H cis), 4.19 – 4.26 (m, 1H, trans), 11.17 (s, 1H, enol). ^13^C NMR (176 MHz, Chloroform-*d*) δ 10.09 (trans), 11.13 (cis), 16.34 (d, *J* = 6.1 Hz, cis), 16.37 (d, *J* = 6.4 Hz, trans), 16.41 (d, *J* = 6.2 Hz, trans), 16.48 (d, *J* = 6.1 Hz, cis), 24.11 (cis), 26.58 (trans), 27.40 (d, *J* = 4.8 Hz, cis), 28.28 (trans), 30.16 (trans), 31.04 (cis), 51.10 (trans), 52.18 (cis), 56.12 (d, *J* = 127.1 Hz, cis), 56.56 – 57.62 (s, trans), 62.26 (d, *J* = 6.7 Hz, cis), 62.41 (d, *J* = 6.8 Hz, trans), 62.52 (d, *J* = 6.8 Hz, cis), 62.99 (d, *J* = 6.8 Hz, trans), 72.50 (d, *J* = 2.2 Hz, trans), 73.81 (d, *J* = 7.4 Hz, cis), 74.92 (trans), 75.38 (cis), 90.22 (d, *J* = 176.1 Hz, enol), 203.18 (cis), 203.33 (d, *J* = 6.3 Hz, trans). ESI-MS [M+Na]^+^ = 315.1. Anal. Calcd for C_13_H_25_O_5_P: C, 53.42; H, 8.62. Found: C, 53.32; H, 8.61.

Due to small amount of the enol form, the signals of enol form in the ^1^H and ^13^C NMR spectra have not been assigned.

Diethyl (2-butyl-6,6-dimethyl-4-oxotetrahydro-2*H*-pyran-3-yl)phosphonate (**10b**) (217,9 mg, 68%) Colorless oil. ^31^P NMR (101 MHz, Chloroform-*d*) δ 19.06 (trans), 19.54 (cis), 26.01(enol). ^1^H NMR (700 MHz, Chloroform-*d*) δ 0.85 – 0.95 (m, 3H trans + 3H cis), 1.16 (s, 3H, cis), 1.20 (s, 3H, trans), 1.27 – 1.36 (m, 13H trans + 10H cis), 1.37 (s, 3H, cis), 1.42 – 1.57 (m, 1H trans + 1H cis), 1.71 – 1.81 (m, 1H, cis), 1.79 – 2.12 (m, 1H, trans), 2.25 (dd, *J* = 13.5, 1.6 Hz, 1H, cis), 2.30 (dd, *J* = 13.2, 1.3 Hz, 1H, trans), 2.80 – 2.89 (m, 2H trans + 1H cis), 2.93 (ddd, *J* = 21.3, 3.4, 1.7 Hz, 1H, cis), 3.90 (dddd, *J* = 38.9, 8.4, 4.7, 3.4 Hz, 1H, cis), 4.06 – 4.21 (m, 4H trans + 4H cis), 4.30 (tdd, *J* = 9.0, 6.6, 2.1 Hz, 1H, trans), 11.18 (s, 1H, enol). ^13^C NMR (176 MHz, Chloroform-*d*) δ 14.14 (cis), 14.17 (trans), 16.40 (d, *J* = 6.2 Hz, trans), 16.45 (d, *J* = 6.2 Hz, trans), 22.54 (cis), 22.57 (trans), 24.12 (cis), 26.69 (trans), 27.96 (trans), 28.84 (cis), 30.21 (trans), 31.10 (cis), 33.92 (d, *J* = 4.3 Hz, cis), 35.09 (trans), 51.09 (trans), 52.22 (cis), 57.59 (d, *J* = 123.3 Hz, trans), 62.28 (d, *J* = 6.9 Hz, cis), 62.42 (d, *J* = 6.7 Hz, trans), 62.55 (d, *J* = 6.9 Hz, cis), 63.03 (d, *J* = 6.8 Hz, trans), 71.32 (d, *J* = 1.9 Hz, trans), 72.26 (d, *J* = 7.3 Hz, cis), 74.98 (trans), 75.43 (cis), 203.37 (trans), 203.40 (cis). ESI-MS [M+Na]^+^ = 343.2. Anal. Calcd for C_15_H_29_O_5_P: C, 56.24; H, 9.12. Found: C, 56.28; H, 9.09.

Due to small amount of the cis and enol form, the signals of this forms in the ^1^H and ^13^C NMR spectra have not been assigned.

Diethyl (2-isopropyl-6,6-dimethyl-4-oxotetrahydro-2*H*-pyran-3-yl)phosphonate (**10c**) (245.1 mg, 80%) Colorless oil. ^31^P NMR (283 MHz, Chloroform-*d*) δ 19.13 (trans), 19.18 (cis), 25.67 (enol). ^1^H NMR (700 MHz, Chloroform-*d*) δ 0.79 (d, *J* = 6.9 Hz, 3H, trans), 0.87 (d, *J* = 6.6 Hz, 3H, cis), 0.93 (d, *J* = 6.6 Hz, 3H, cis), 0.95 (d, *J* = 6.9 Hz, 3H, trans), 1.08 (s, 3H, cis), 1.12 (s, 3H, trans), 1.22 (s, 3H, trans), 1.23 – 1.27 (m, 6H trans + 6H cis), 1.30 (s, 3H, cis), 1.94 (heptd, *J* = 6.6, 1.5 Hz, 1H, cis), 2.05 (heptd, *J* = 6.9, 2.1 Hz, 1H, trans), 2.15 – 2.19 (m, 1H, cis), 2.21 (dd, *J* = 13.1, 1.1 Hz, 1H, trans), 2.73 (d, *J* = 13.1 Hz, 1H, trans), 2.81 (d, *J* = 13.6 Hz, 1H, cis), 2.95 (ddd, *J* = 23.2, 10.0, 1.2 Hz, 1H, trans), 3.05 (ddd, *J* = 20.4, 3.0, 1.6 Hz, 1H, cis), 3.31 (ddd, *J* = 39.5, 10.1, 3.0 Hz, 1H, cis), 3.96 – 4.12 (m, 4H trans + 4H cis), 4.15 (ddd, *J* = 10.1, 6.7, 2.1 Hz, 1H, trans), 11.06 (s, 1H, enol). ^13^C NMR (176 MHz, Chloroform-*d*) δ 13.98 (trans), 16.16 (d, *J* = 6.3 Hz, cis), 16.21 (d, *J* = 6.2 Hz, trans), 16.28 (d, *J* = 6.2 Hz, trans), 16.35 (d, *J* = 6.5 Hz, cis), 18.73 (cis), 20.40 (trans), 20.57 (cis), 24.07 (cis), 26.07 (trans), 29.96 (trans), 30.28 (trans), 30.88 (cis), 31.34 (d, *J* = 4.3 Hz, cis), 51.06 (trans), 51.84 (cis), 54.51 (d, *J* = 124.8 Hz, trans), 55.38 (d, *J* = 127.9 Hz, cis), 62.05 (d, *J* = 6.4 Hz, cis), 62.17 (d, *J* = 6.7 Hz, trans), 62.42 (d, *J* = 6.8 Hz, cis), 62.88 (d, *J* = 6.7 Hz, trans), 74.37 (trans), 74.62 (d, *J* = 2.8 Hz, trans), 75.03 (cis), 78.21 (d, *J* = 7.1 Hz, cis), 90.09 (d, *J* = 176.7 Hz, enol), 203.29 (d, *J* = 2.1 Hz, cis), 203.54 (d, *J* = 6.3 Hz, trans). ESI-MS [M+Na]^+^ = 329.1. Anal. Calcd for C_14_H_27_O_5_P: C, 54.89; H, 8.88. Found: C, 54.95; H, 8.89.

Due to small amount of the enol form, the signals of enol form in the ^1^H and ^13^C NMR spectra have not been assigned.

Diethyl (2-ethyl-4-oxo-1-oxaspiro[5.5]undecan-3-yl)phosphonate (**10e**) (242.6 mg, 73%) Colourless oil. ^31^P NMR (101 MHz, Chloroform-*d*) δ 19.38 (trans), 19.60 (cis), 26.22 (enol). ^1^H NMR (700 MHz, Chloroform-*d*) δ 1.01 (t, *J* = 7.4 Hz, 3H trans + 3H cis), 1.28 – 1.32 (m, 6H trans + 6H cis), 1.09 – 1.91 (m, 11H trans + 11H cis), 1.98 (dqd, *J* = 14.6, 7.4, 2.6 Hz, 1H trans + 1H cis), 2.23 (dd, *J* = 13.4, 1.6 Hz, 1H cis), 2.29 (dd, *J* = 13.4, 1.2 Hz, 1H trans), 2.70 (d, *J* = 13.4 Hz, 1H trans), 2.76 (d, *J* = 13.4 Hz, 1H cis), 2.83 (ddd, *J* = 23.2, 9.8, 1.2 Hz, 1H trans), 2.93 (ddd, *J* = 21.4, 3.5, 1.6 Hz, 1H cis), 3.77 (ddt, *J* = 38.6, 9.3, 3.9 Hz, 1H cis), 4.06 – 4.16 (m, 4H trans + 4H cis), 4.19 (tdd, *J* = 9.2, 6.7, 2.4 Hz, 1H trans), 11.17 (d, *J* = 1.1 Hz, 1H enol). ^13^C NMR (176 MHz, Chloroform-*d*) δ 10.51 (trans), 11.53 (cis), 16.35 (d, *J* = 6.1 Hz, trans), 16.42 (d, *J* = 6.0 Hz, trans), 21.23 (cis), 21.75 (cis), 21.80 (trans), 21.91 (trans), 25.43 (trans), 25.53 (cis), 27.60 (d, *J* = 4.5 Hz, cis), 28.76 (trans), 31.62 (cis), 34.61 (trans), 39.12 (trans), 39.56 (cis), 50.85 (trans), 52.00 (cis), 56.65 (d, *J* = 127.4 Hz, cis), 57.53 (d, *J* = 124.3 Hz, trans), 62.20 (d, *J* = 6.4 Hz, cis), 62.32 (d, *J* = 6.8 Hz, trans), 62.51 (d, *J* = 6.4 Hz, cis), 62.96 (d, *J* = 6.8 Hz, trans), 71.77 (d, *J* = 2.3 Hz, trans), 72.51 (d, *J* = 7.3 Hz, cis), 75.72 (trans), 76.30 (cis), 203.27 (d, *J* = 1.9 Hz, cis), 203.41 (d, *J* = 6.4 Hz, trans). ESI-MS [M+Na]^+^ = 355.2. Anal. Calcd for C_16_H_29_O_5_P: C, 57.82; H, 8.79. Found: C, 57,92; H, 8.77.

Due to small amount of the cis and enol form, the signals of cis and enol forms in the ^1^H and ^13^C NMR spectra have not been assigned.

Diethyl (2-butyl-4-oxo-1-oxaspiro[5.5]undecan-3-yl)phosphonate (**10f**) (255.9 mg, 71%) Colourless oil. ^31^P NMR (101 MHz, Chloroform-*d*) δ 19.30 (trans), 19.66 (cis), 26.22 (enol). ^1^H NMR (700 MHz, Chloroform-*d*) δ 0.87 (t, *J* = 7.2 Hz, 3H trans + 3H cis), 1.12 – 2.02 (m, 22H trans + 22H cis), 2.21 (dd, *J* = 13.3, 1.7 Hz, 1H, cis), 2.27 (dd, *J* = 13.5, 1.3 Hz, 1H, trans), 2.69 (d, *J* = 13.4 Hz, 1H, trans), 2.73 (d, *J* = 13.4 Hz, 1H, cis), 2.80 (ddd, *J* = 23.3, 9.7, 1.2 Hz, 1H, trans), 2.89 (ddd, *J* = 21.4, 3.5, 1.7 Hz, 1H, cis), 3.84 (ddt, *J* = 38.7, 9.4, 3.7 Hz, 1H, cis), 4.03 – 4.16 (m, 4H trans + 4H cis), 4.25 (tdd, *J* = 9.5, 6.9, 2.2 Hz, 1H, trans), 11.14 (d, *J* = 1.2 Hz, 1H, enol). ^13^C NMR (176 MHz, Chloroform-*d*) δ 14.07 (trans), 16.31 (d, *J* = 6.2 Hz, trans), 16.37 (d, *J* = 6.3 Hz, trans), 21.25 (cis), 21.74 (cis), 21.80 (trans), 21.89 (trans), 22.47 (trans), 22.54 (cis), 25.39 (trans), 25.50 (cis), 28.09 (trans), 29.03 (cis), 31.60 (cis), 34.01 (d, *J* = 4.7 Hz, cis), 34.71 (trans), 35.37 (trans), 39.05 (trans), 39.53 (cis), 50.69 (trans), 51.91 (cis), 56.79 (d, *J* = 125.7 Hz, cis), 57.85 (d, *J* = 123.5 Hz, trans), 62.14 (d, *J* = 6.8 Hz, cis), 62.26 (d, *J* = 6.6 Hz, trans), 62.46 (d, *J* = 6.8 Hz, cis), 62.91 (d, *J* = 6.7 Hz, trans), 70.44 (d, *J* = 2.0 Hz, trans), 71.02 (d, *J* = 7.1 Hz, cis), 75.74 (trans), 76.33 (cis), 203.23 (d, *J* = 1.1 Hz, cis), 203.35 (d, *J* = 6.4 Hz, trans). ESI-MS [M+Na]^+^ = 383.2. Anal. Calcd for C_18_H_33_O_5_P: C, 59.98; H, 9.23. Found: C, 60.13 ; H, 9.21.

Due to small amount of the cis and enol form, the signals of cis and enol forms in the ^1^H and ^13^C NMR spectra have not been assigned.

Diethyl (2-isopropyl-4-oxo-1-oxaspiro[5.5]undecan-3-yl)phosphonate (**10g**) (259.8 mg, 75%) Colourless oil.  ^31^P NMR (101 MHz, Chloroform-*d*) δ 19.64 (cis), 19.75 (trans), 26.06 (enol). ^1^H NMR (700 MHz, Chloroform-*d*) δ 0.86 (d, *J* = 6.8 Hz, 3H, trans), 0.92 (d, *J* = 6.5 Hz, 3H, cis), 1.04 (d, *J* = 6.9 Hz, 3H trans + 3H cis), 1.09 – 2.01 (m, 16H trans + 16H cis), 2.09 (heptd, *J* = 6.7, 1.9 Hz, 1H, trans), 2.19 (dd, *J* = 13.4, 1.7 Hz, 1H, cis), 2.24 (dd, *J* = 13.3, 1.2 Hz, 1H, trans), 2.29 (dhept, *J* = 10.1, 6.5 Hz, 1H, cis), 2.66 (d, *J* = 13.3 Hz, 1H, trans), 2.78 (d, *J* = 13.5 Hz, 1H, cis), 3.00 (ddd, *J* = 23.0, 10.0, 1.2 Hz, 1H, trans), 3.10 (ddd, *J* = 20.3, 3.2, 1.6 Hz, 1H, cis), 3.32 (ddd, *J* = 39.2, 10.2, 3.1 Hz, 1H, cis), 3.94 – 4.16 (m, 4H trans + 4H cis), 4.18 (ddd, *J* = 9.6, 7.0, 2.1 Hz, 1H, trans), 11.10 (s, 1H, enol). ^13^C NMR (176 MHz, Chloroform-*d*) δ 14.19 (trans), 16.30 (d, *J* = 6.4 Hz, trans), 16.38 (d, *J* = 6.4 Hz, trans), 18.97 (cis), 20.89 (cis), 20.94 (trans), 21.14 (cis), 21.71 (trans), 21.80 (cis), 21.83 (trans), 25.42 (trans), 25.48 (cis), 30.78 (trans), 31.48 (cis), 31.78 (d, *J* = 4.5 Hz, cis), 34.11 (trans), 39.19 (trans), 39.62 (cis), 51.06 (trans), 51.89 (cis), 54.82 (d, *J* = 125.6 Hz, trans), 55.79 (d, *J* = 127.0 Hz, cis), 62.10 (d, *J* = 6.5 Hz, cis), 62.19 (d, *J* = 6.8 Hz, trans), 62.51 (d, *J* = 6.7 Hz, cis), 62.95 (d, *J* = 6.9 Hz, trans), 74.04 (d, *J* = 2.8 Hz, trans), 75.22 (trans), 76.27 (cis), 77.11 (cis), 90.16 (d, *J* = 176.3 Hz, enol), 203.49 (d, *J* = 2.4 Hz, cis), 203.74 (d, *J* = 6.3 Hz, trans). ESI-MS [M+Na]^+^ = 369.1. Anal. Calcd for C_17_H_31_O_5_P: C, 58.94; H, 9.02. Found: C, 58.90; H, 9.04.

Due to small amount of the cis and enol form, the signals of cis and enol forms in the ^1^H and ^13^C NMR spectra have not been assigned.

Diethyl (2-ethyl-4-oxo-6,6-diphenyltetrahydro-2*H*-pyran-3-yl)phosphonate (**10i**) (291.5 mg, 70%) Pale yellow oil. ^31^P NMR (101 MHz, Chloroform-*d*) δ 19.25 (trans), 19.37 (cis), 25.52 (enol). ^1^H NMR (700 MHz, Chloroform-*d*) δ 0.90 (t, *J* = 7.1 Hz, 3H, trans), 1.00 (t, *J* = 7.4 Hz, 3H, enol), 1.11 (t, *J* = 7.3 Hz, 3H, cis), 1.14 (t, *J* = 7.3 Hz, 3H, trans), 1.26 (t, *J* = 7.1 Hz, 3H, cis), 1.30 – 1.32 (m, 3H, enol), 1.33 – 1.36 (m, 3H trans + 3H cis + 3H enol), 1.71 (dt, *J* = 14.1, 7.1 Hz, 1H, cis), 1.82 (dddd, *J* = 14.1, 7.2, 6.3, 1.9 Hz, 2H, enol), 1.97 (dddd, *J* = 14.0, 7.3, 3.1, 0.7 Hz, 1H, cis), 2.11 – 2.18 (m, 1H, trans), 2.16 – 2.25 (m, 1H, trans), 2.75 (ddd, *J* = 17.1, 4.4, 2.7 Hz, 1H, enol), 2.88 (ddq, *J* = 10.1, 8.2, 7.1 Hz, 1H, enol), 2.98 (ddd, *J* = 22.2, 3.6, 1.6 Hz, 1H, cis), 3.00 (dd, *J* = 13.5, 0.9 Hz, 1H, trans), 3.11 (ddd, *J* = 22.4, 10.0, 1.1 Hz, 1H, trans), 3.17 – 3.25 (m, 1H cis + 1H enol), 3.45 (ddd, *J* = 14.5, 1.7, 0.8 Hz, 1H, cis), 3.49 (dtd, *J* = 10.1, 7.1, 6.5 Hz, 1H, enol), 3.64 (ddt, *J* = 39.0, 9.3, 4.1 Hz, 1H, cis), 3.74 (dd, *J* = 13.8, 1.0 Hz, 1H, trans), 3.81 – 3.85 (m, 1H, trans), 4.01 – 4.31 (m, 4H trans + 4H cis + 3H enol), 7.14 – 7.48 (m, 10H trans + 10H cis + 10H enol), 11.37 (s, 1H, enol). ^13^C NMR (176 MHz, Chloroform-*d*) δ 9.18 (enol), 10.00 (trans), 11.15 (cis), 15.89 (d, *J* = 7.2 Hz, enol), 16.16 (d, *J* = 7.0 Hz, enol), 16.21 – 16.33 (2$\times$C cis + trans), 16.36 (d, *J* = 6.6 Hz, trans), 27.24 (d, *J* = 4.5 Hz, cis), 27.90 (trans), 28.49 (enol), 39.32 (d, *J* = 12.1 Hz, enol), 50.84 (cis), 51.28 (trans), 55.72 (d, *J* = 129.2 Hz, trans), 56.40 (d, *J* = 126.3 Hz, cis), 61.41 (d, *J* = 3.6 Hz, enol), 61.82 (d, *J* = 5.0 Hz, enol), 62.21 (d, *J* = 6.4 Hz, trans), 62.42 (d, *J* = 6.6 Hz, cis), 62.47 (d, *J* = 7.0 Hz, cis), 62.86 (d, *J* = 6.6 Hz, trans), 70.38 (d, *J* = 15.3 Hz, enol), 72.97 (d, *J* = 3.1 Hz, trans), 73.63 (d, *J* = 7.3 Hz, cis), 78.13 (enol), 81.82 (trans), 83.08 (cis), 90.32 (d, *J* = 173.7 Hz, enol), 125.02 (trans, 2$\times$C), 125.20 (enol, 2$\times$C), 125.26 (cis, 2$\times$C), 126.78 (enol), 127.02 (trans), 127.08 (cis), 127.10 (trans, 2$\times$C), 127.22 (enol, 2$\times$C), 127.25 (trans), 127.81 (enol), 127.97 (cis), 128.09 (cis, 2$\times$C), 128.11 (cis, 2$\times$C), 128.13 (trans, 2$\times$C), 128.19 (enol, 2$\times$C), 128.37 (trans, 2$\times$C), 128.51 (enol, 2$\times$C), 128.57 (cis, 2$\times$C), 142.05 (enol), 142.06 (cis) 143.60 (cis), 146.87 (trans), 147.19 (enol), 147.34 (trans), 168.47 (d, *J* = 3.8 Hz, enol), 201.40 (d, *J* = 2.6 Hz, cis), 201.75 (d, *J* = 6.8 Hz, trans). ESI-MS [M+Na]^+^ = 439.1. Anal. Calcd for C_23_H_29_O_5_P: C, 66.33; H, 7.02. Found: C, 66.18; H, 7.04.

Diethyl (2-butyl-4-oxo-6,6-diphenyltetrahydro-2*H*-pyran-3-yl)phosphonate (**10j**) (333.4 mg, 75%) Pale yellow oil. ^31^P NMR (101 MHz, Chloroform-*d*) δ 19.23 (trans), 19.44 (cis), 25.56 (enol). ^1^H NMR (700 MHz, Chloroform-*d*) δ 0.87 – 0.92 (m, 3H trans + 3H cis ), 0.94 (t, *J* = 7.4 Hz, 3H, enol), 0.98 (t, *J* = 7.4 Hz, 3H, cis), 1.11 – 2.31 (m, 12H trans + 9H cis + 12H enol), 2.75 (ddd, *J* = 17.1, 4.4, 2.7 Hz, 1H, enol), 2.89 (ddq, *J* = 10.1, 8.2, 7.1 Hz, 1H, enol) 2.93 – 3.03 (m, 1H trans + 1H cis), 3.09 (ddd, *J* = 22.4, 10.0, 1.1 Hz, 1H, trans), 3.17 – 3.24 (m, 1H cis + 1H enol), 3.44 (ddd, *J* = 14.4, 1.7, 0.8 Hz, 1H, cis), 3.51 (m, 1H, enol), 3.65 – 3.80 (m, 1H trans + 1H cis), 3.81 – 3.88 (m, 1H, trans), 4.01 – 4.31 (m, 4H trans + 4H cis + 3H enol), 7.16 – 7.48 (m, 10H trans + 10H cis + 10H enol), 11.36 (s, 1H, enol). ^13^C NMR (176 MHz, Chloroform-*d*) δ 14.03 (cis), 14.09 (enol), 14.28 (trans), 15.93 (d, *J* = 7.4 Hz, enol), 16.22 (d, *J* = 7.1 Hz, trans), 16.27 – 16.38 (3$\times$C, trans + cis + enol), 16.42 (d, *J* = 6.5 Hz, cis), 22.42 (cis), 22.57 (trans), 22.78 (enol), 27.04 (trans), 27.62 (enol), 28.54 (cis), 33.74 (d, *J* = 4.3 Hz, cis), 34.77 (enol), 35.38 (cis), 39.29 (d, *J* = 12.1 Hz, enol), 50.91 (cis), 51.29 (trans), 56.62 (d, *J* = 128.9 Hz, trans), 56.69 (d, *J* = 126.4 Hz, cis), 61.48 (d, *J* = 3.7 Hz, trans), 61.82 (d, *J* = 4.9 Hz, enol), 62.24 (d, *J* = 6.6 Hz, cis), 62.45 (d, *J* = 6.4 Hz, enol), 62.52 (d, *J* = 6.8 Hz, cis), 62.90 (d, *J* = 6.9 Hz, trans), 69.41 (d, *J* = 15.3 Hz, enol), 71.90 (d, *J* = 3.1 Hz, trans), 72.11 (d, *J* = 7.2 Hz, cis), 78.23 (enol), 81.92 (trans), 83.17 (cis), 90.73 (d, *J* = 173.6 Hz, enol), 125.06 (trans, 2$\times$C), 125.29 (enol, 2$\times$C), 125.34 (cis, 2$\times$C), 126.83 (trans), 127.08 (cis), 127.13 (enol), 127.18 (trans, 2$\times$C), 127.28 (enol, 2$\times$C), 127.32 (enol), 127.87 (trans), 128.02 (cis), 128.17 (cis, 4$\times$C), 128.18 (trans, 3$\times$C), 128.24 (enol, 2$\times$C), 128.40 (enol, 2$\times$C), 128.52 (trans, 2$\times$C), 128.59 (cis, 2$\times$C), 142.08 (enol), 142.15 (cis), 143.69 (trans), 146.92 (cis), 147.26 (enol), 147.41 (trans), 168.30 (d, *J* = 3.8 Hz, enol), 201.53 (d, *J* = 2.6 Hz, cis), 201.81 (d, *J* = 6.8 Hz, trans). ESI-MS [M+Na]^+^ = 467.1. Anal. Calcd for C_25_H_33_O_5_P: C, 67.55; H, 7.48. Found: C, 67.33; H, 7.46.

Diethyl (2-isopropyl-4-oxo-6,6-diphenyltetrahydro-2*H*-pyran-3-yl)phosphonate (**10k**) (284.1 mg, 66%) Pale yellow oil. ^31^P NMR (101 MHz, Chloroform-*d*) δ 19.31 (cis) , 19.63 (trans), 25.53 (enol). ^1^H NMR (700 MHz, Chloroform-*d*) δ 0.89 (t, *J* = 7.0 Hz, 3H, enol), 1.02 (d, *J* = 6.7 Hz, 3H, enol), 1.11 (d, *J* = 6.9 Hz, 3H, trans), 1.18 (d, *J* = 6.8 Hz, 3H, trans), 1.23 (d, *J* = 6.9 Hz, 3H, enol), 1.27 (t, *J* = 7.1 Hz, 3H, trans), 1.31 (t, *J* = 7.1 Hz, 3H, trans), 1.33 (t, *J* = 7.2 Hz, 3H, enol), 2.11 (heptd, *J* = 6.8, 2.1 Hz, 1H, enol), 2.29 (heptd, *J* = 6.8, 1.9 Hz, 1H, trans), 2.71 (ddd, *J* = 16.9, 4.5, 2.7 Hz, 1H, enol), 2.74 – 2.80 (m, 2H, trans), 2.90 (ddd, *J* = 13.7, 1.1, 0.5 Hz, 1H, trans), 3.19 (ddd, *J* = 16.9, 1.9, 1.1 Hz, 1H, enol), 3.26 (ddd, *J* = 21.6, 10.2, 1.0 Hz, 1H, trans), 3.41 – 3.48 (m, 2H, trans), 3.74 (dd, *J* = 13.7, 1.7 Hz, 1H, trans), 3.77 (m, 1H, enol), 4.02 (ddd, *J* = 10.3, 5.3, 1.9 Hz, 1H, trans), 4.04 – 4.19 (m, 4H, enol), 7.15 – 7.48 (m, 10H enol + 10H trans), 11.38 (s, 1H, enol). ^13^C NMR (176 MHz, Chloroform-*d*) δ 14.76 (trans), 15.26 (enol), 16.01 (d, *J* = 7.5 Hz, enol), 16.28 (d, *J* = 7.5 Hz, enol), 16.33 (d, *J* = 6.7 Hz, trans), 16.43 (d, *J* = 6.6 Hz, trans), 20.18 (enol), 21.07 (trans), 31.15 (trans), 31.83 (enol), 39.55 (d, *J* = 12.2 Hz, enol), 51.58 (trans), 54.16 (d, *J* = 132.0 Hz, trans), 55.06 (d, *J* = 126.5 Hz, cis), 61.43 (d, *J* = 3.6 Hz, enol), 62.00 (d, *J* = 5.1 Hz, enol), 62.07 (d, *J* = 6.9 Hz, trans), 62.98 (d, *J* = 6.8 Hz, trans), 74.04 (d, *J* = 15.8 Hz, enol), 75.68 (d, *J* = 3.9 Hz, trans), 77.93 (enol), 81.79 (trans), 90.33 (d, *J* = 173.7 Hz, enol), 125.07 (enol, 2$\times$C), 125.14 (trans, 2$\times$C), 126.83 (enol), 127.10 (trans), 127.21 (enol, 2$\times$C), 127.28 (trans, 2$\times$C), 127.61 (enol), 127.97 (trans), 128.23 (enol, 2$\times$C), 128.30 (trans, 2$\times$C), 128.50 (enol, 2$\times$C), 128.52 (trans, 2$\times$C), 142.25 (enol), 143.11 (trans), 147.41 (enol + trans, 2$\times$C), 168.80 (d, *J* = 3.8 Hz, enol), 202.22 (d, *J* = 6.7 Hz, trans). ESI-MS [M+Na]^+^ = 453.1. Anal. Calcd for C_24_H_31_O_5_P: C, 66.96; H, 7.26. Found: C, 66.80; H, 7.24.

Due to small amount of the cis form, the signals of cis form in the ^1^H and ^13^C NMR spectra have not been assigned.

Diethyl (6'-ethyl-4'-oxo-3',4',5',6'-tetrahydrospiro[fluorene-9,2'-pyran]-5'-yl)phosphonate (**10m**) (339.8 mg, 82%) Yellow oil. ^31^P NMR (101 MHz, Chloroform-*d*) δ 18.66 (trans), 19.78 (cis), 24.37 (enol). ^1^H NMR (700 MHz, Chloroform-*d*) δ 0.77 (t, *J* = 7.4 Hz, 3H, cis), 0.82 (t, *J* = 7.3 Hz, 3H, trans), 0.84 (t, *J* = 7.2 Hz, 3H, enol), 1.28 – 1.39 (m, 6H trans + 6H cis + 6H enol), 1.55 (ddq, *J* = 14.4, 8.4, 7.2 Hz, 1H, trans), 1.65 (dqd, *J* = 14.4, 7.2, 5.6 Hz, 1H, enol), 1.75 – 1.87 (m, 2H cis + 1H enol), 1.94 – 2.04 (m, 1H, trans), 2.12 (dt, *J* = 17.1, 1.2 Hz, 1H, enol), 2.36 (ddd, *J* = 13.8, 1.8, 0.8 Hz, 1H, cis), 2.60 (dd, *J* = 13.8, 1.3 Hz, 1H, trans), 3.02 (ddd, *J* = 17.1, 4.2, 2.5 Hz, 1H, enol), 3.19 (ddd, *J* = 23.7, 10.1, 1.2 Hz, 1H, trans), 3.29 (ddd, *J* = 21.7, 3.4, 1.7 Hz, 1H, cis), 3.53 (d, *J* = 13.7 Hz, 1H, trans), 3.65 (d, *J* = 13.9 Hz, 1H, cis), 3.99 – 4.26 (m, 4H trans + 4H cis + 4H enol), 4.38 (dtd, *J* = 38.6, 6.8, 3.4 Hz, 1H, cis), 4.51 (ddtd, *J* = 5.7, 3.5, 2.2, 1.3 Hz, 1H, enol), 4.84 – 4.95 (m, 1H, trans), 7.08 – 7.77 (m, 8H trans + 8H cis + 8H enol), 11.30 (s, 1H, enol). ^13^C NMR (176 MHz, Chloroform-*d*) δ 8.40 (enol), 9.76 (trans), 10.67 (cis), 16.30 (d, *J* = 6.9 Hz, cis), 16.40 (d, *J* = 6.4 Hz, trans), 16.48 (d, *J* = 6.3 Hz, trans), 16.66 (d, *J* = 6.0 Hz, cis), 27.51 (d, *J* = 4.8 Hz, cis), 27.57 (enol), 28.34 (trans), 36.76 (d, *J* = 12.2 Hz, enol), 46.59 (trans), 47.70 (cis), 56.71 (d, *J* = 127.7 Hz, cis), 57.52 (d, *J* = 123.2 Hz, trans), 62.18 (d, *J* = 6.3 Hz, enol), 62.44 (d, *J* = 5.1 Hz, enol), 62.59 (d, *J* = 6.7 Hz, cis), 62.72 (d, *J* = 6.6 Hz, trans), 62.87 (d, *J* = 7.0 Hz, cis), 63.28 (d, *J* = 6.9 Hz, trans), 71.89 (d, *J* = 14.3 Hz, cis), 74.67 (d, *J* = 2.4 Hz, trans), 76.13 (d, *J* = 7.0 Hz, enol), 80.43 (enol), 83.87 (trans), 84.58 (cis), 91.86 (d, *J* = 178.6 Hz, enol), 119.84 (cis), 119.87 (enol), 119.97 (trans), 120.44 (trans), 120.46 (enol), 120.74 (cis), 123.89 (trans), 123.96 (trans), 124.00 (cis), 124.48 (cis), 124.70 (enol), 125.20 (enol), 127.31 (enol), 127.60 (enol), 128.09 (trans), 128.35 (cis), 128.48 (trans), 128.57 (cis), 129.26 (enol), 129.42 (enol), 129.49 (cis), 129.52 (trans), 129.59 (trans), 129.70 (cis), 138.66 (trans), 138.96 (enol), 139.42 (cis), 139.88 (trans), 140.33 (enol), 140.49 (cis), 144.82 (cis), 145.48 (enol), 146.57 (cis), 146.73 (trans), 147.38 (enol), 147.67 (trans), 167.83 (d, *J* = 4.0 Hz, enol), 202.40 (d, *J* = 6.5 Hz, trans), 202.52 (cis). ESI-MS [M+Na]^+^ = 437.1. Anal. Calcd for C_23_H_27_O_5_P: C, 66.66; H, 6.57. Found: C, 66.52; H, 6.56.

Due to small amount of the enol form, the signals of enol form in the ^1^H and ^13^C NMR spectra have not been assigned.

Diethyl (6'-butyl-4'-oxo-3',4',5',6'-tetrahydrospiro[fluorene-9,2'-pyran]-5'-yl)phosphonate (**10n**) (269.9 mg, 61%). Yellow oil. ^31^P NMR (101 MHz, Chloroform-*d*) δ 18.62 (trans), 19.86 (cis), 24.48 (enol). ^1^H NMR (700 MHz, Chloroform-*d*) δ 0.67 – 0.76 (m, 3H trans + 3H cis), 0.79 (t, *J* = 7.3 Hz, 3H, enol), 1.07 – 1.25 (m, 2H trans + 2H cis + 3H enol), 1.25 – 1.40 (m, 8H trans + 8H cis + 8H enol), 1.51 (dtd, *J* = 13.9, 9.0, 4.6 Hz, 1H, trans), 1.57 – 1.66 (m, 1H, enol), 1.71 – 1.87 (m, 2H, cis), 1.89 – 1.99 (m, 1H, trans), 2.13 (m, 1H, enol), 2.36 (dd, *J* = 13.9, 1.7 Hz, 1H, cis), 2.58 (d, *J* = 13.7 Hz, 1H, trans), 3.02 (ddd, *J* = 17.0, 4.1, 2.5 Hz, 1H, enol), 3.18 (dd, *J* = 23.7, 10.0 Hz, 1H, trans), 3.26 (ddd, *J* = 21.6, 3.5, 1.7 Hz, 1H, cis), 3.56 (d, *J* = 13.7 Hz, 1H, trans), 3.65 (d, *J* = 13.8 Hz, 1H, cis), 3.99 – 4.26 (m, 4H trans + 4H cis + 4H enol), 4.47 (dddd, *J* = 38.6, 8.1, 5.8, 3.3 Hz, 1H, cis), 4.53 (dd, *J* = 6.0, 3.0 Hz, 1H, enol), 4.96 (tdd, *J* = 9.2, 6.8, 2.1 Hz, 1H, trans), 7.04 – 7.75 (m, 8H trans + 8H cis + 8H enol), 11.28 (s, 1H, enol). ^13^C NMR (176 MHz, Chloroform-*d*) δ 13.92 (cis), 13.96 (trans), 14.24 (enol), 16.32 (d, *J* = 7.0 Hz, cis), 16.42 (d, *J* = 6.3 Hz, trans), 16.49 (d, *J* = 6.3 Hz, trans), 16.66 (d, *J* = 5.8 Hz, cis), 22.40 (enol), 22.45 (trans), 22.89 (cis), 26.25 (enol), 27.42 (trans), 28.13 (cis), 34.04 (d, *J* = 4.6 Hz, cis), 34.58 (enol), 35.22 (trans), 36.75 (d, *J* = 12.1 Hz, enol), 46.54 (trans), 47.70 (cis), 57.06 (d, *J* = 127.5 Hz, cis), 58.13 (d, *J* = 122.4 Hz, trans), 62.18 (d, *J* = 6.4 Hz, enol), 62.37 (d, *J* = 5.0 Hz, enol), 62.55 (d, *J* = 6.5 Hz, cis), 62.69 (d, *J* = 6.5 Hz, trans), 62.87 (d, *J* = 6.9 Hz, cis), 63.27 (d, *J* = 6.9 Hz, trans), 71.23 (d, *J* = 14.1 Hz, cis), 73.58 (d, *J* = 2.1 Hz, trans), 74.59 (d, *J* = 7.1 Hz, enol), 80.49 (cis), 83.89 (trans), 84.59 (enol), 92.26 (d, *J* = 178.7 Hz, enol), 119.83 (cis), 119.86 (enol), 119.96 (trans), 120.43 (trans), 120.46 (cis), 120.75 (enol), 123.84 (trans), 123.99 (trans), 124.05 (enol), 124.50 (cis), 124.69 (enol), 125.14 (cis), 127.31 (cis), 127.59 (enol), 128.10 (trans), 128.34 (enol), 128.48 (trans), 128.57 (cis), 129.25 (cis), 129.41 (enol), 129.49 (cis), 129.50 (trans), 129.59 (trans), 129.69 (enol), 138.64 (trans), 138.95 (enol), 139.43 (cis), 139.87 (trans), 140.32 (enol), 140.48 (cis), 144.87 (enol), 145.45 (cis), 146.59 (enol), 146.82 (trans), 147.39 (cis), 147.73 (trans), 167.62 (d, *J* = 3.9 Hz, enol), 202.45 (d, *J* = 6.6 Hz, trans), 202.58 (cis) ). ESI-MS [M+Na]^+^ = 465.1. Anal. Calcd for C_25_H_31_O_5_P: C, 67.86; H, 7.06. Found: C, 67.99; H, 7.07.

Due to small amount of the enol form, the signals of enol form in the ^1^H and ^13^C NMR spectra have not been assigned.

Diethyl (6'-isopropyl-4'-oxo-3',4',5',6'-tetrahydrospiro[fluorene-9,2'-pyran]-5'-yl)phosphonate (**10o**) (171.4 mg, 40%) Yellow oil. ^31^P NMR (101 MHz, Chloroform-*d*) δ 18.85 (trans), 19.94 (cis), 24.36 (enol). ^1^H NMR (700 MHz, Chloroform-*d*) δ 0.64 (d, *J* = 6.4 Hz, 3H, cis), 0.81 (d, *J* = 6.8 Hz, 3H, trans), 0.83 (d, *J* = 6.9 Hz, 3H, enol), 0.84 (d, *J* = 6.9 Hz, 3H, trans), 0.90 (d, *J* = 6.6 Hz, 3H, enol), 0.91 (d, *J* = 6.6 Hz, 3H, cis), 1.28 – 1.42 (m, 6H trans + 6H cis + 6H enol), 2.05 – 2.11 (m, 2H, enol), 2.19 (m, 1H trans + 1H cis), 2.38 (ddd, *J* = 14.0, 1.8, 0.7 Hz, 1H, cis), 2.56 (dd, *J* = 13.6, 1.3 Hz, 1H, trans), 2.97 (ddd, *J* = 16.9, 4.5, 2.5 Hz, 1H, enol), 3.36 (ddd, *J* = 23.8, 10.2, 1.2 Hz, 1H, trans), 3.43 (ddd, *J* = 20.7, 3.0, 1.7 Hz, 1H, cis), 3.54 (d, *J* = 13.7 Hz, 1H, trans), 3.69 (d, *J* = 14.0 Hz, 1H, cis), 3.98 (ddd, *J* = 39.2, 10.1, 2.9 Hz, 1H, cis), 4.03 – 4.26 (m, 4H trans + 4H cis + 4H enol ), 4.40 (dd, *J* = 2.4, 1.3 Hz, 1H, enol), 4.92 (ddd, *J* = 10.3, 7.2, 2.0 Hz, 1H, trans), 7.09 – 7.79 (m, 8H trans + 8H cis + 8H enol ), 11.26 (s, 1H, enol). ^13^C NMR (176 MHz, Chloroform-*d*) δ 13.89 (trans), 14.83 (enol), 16.37 (d, *J* = 6.8 Hz, enol), 16.46 (d, *J* = 6.4 Hz, trans), 16.53 (d, *J* = 6.0 Hz, trans), 16.71 (d, *J* = 5.9 Hz, cis), 18.85 (cis), 20.07 (enol), 20.49 (trans), 30.83 (trans), 31.83 (d, *J* = 4.2 Hz, cis), 31.88 (enol), 37.32 (d, *J* = 12.5 Hz, enol), 46.73 (trans), 47.54 (cis), 55.48 (d, *J* = 126.5 Hz, cis), 55.55 (d, *J* = 123.3 Hz, trans), 62.21 (d, *J* = 6.9 Hz, enol), 62.54 (d, *J* = 5.8 Hz, enol), 62.56 (d, *J* = 5.2 Hz, cis), 62.71 (d, *J* = 6.5 Hz, trans), 62.91 (d, *J* = 7.0 Hz, cis), 63.30 (d, *J* = 7.0 Hz, trans), 75.64 (d, *J* = 14.4 Hz, cis), 77.01 (trans), 80.27 (enol), 80.58 (cis), 83.70 (trans), 92.20 (d, *J* = 178.5 Hz, enol), 119.79 (cis), 119.83 (enol), 119.97 (trans), 120.45 (trans), 120.46 (enol), 120.73 (cis), 123.96 (trans), 124.03 (trans), 124.04 (enol), 124.63 (cis), 124.74 (enol), 125.24 (cis), 127.30 (cis), 127.68 (enol), 128.10 (trans), 128.25 (enol), 128.41 (trans), 128.54 (cis), 129.17 (enol), 129.30 (cis), 129.44 (trans), 129.46 (enol), 129.55 (trans), 129.64 (cis), 138.64 (trans), 139.06 (enol), 139.36 (cis), 139.88 (trans), 140.33 (cis), 140.50 (enol), 145.11 (cis), 145.85 (enol), 146.81 (enol), 146.96 (trans), 147.83 (cis), 147.98 (trans), 167.71 (d, *J* = 4.0 Hz, enol), 202.71 (d, *J* = 6.7 Hz, trans). ESI-MS [M+Na]^+^ = 451.1. Anal. Calcd for C_24_H_29_O_5_P: C, 67.28; H, 6.82. Found: C, 67.34; H, 6.81.

Due to small amount of the cis and enol forms, the signals of cis and enol forms in the ^1^H and ^13^C NMR spectra have not been assigned.

2.5 Synthesis of diethyl (2-aryl-6,6-alkyl(aryl)-4-oxotetrahydro-2H-pyran-3-yl)phosphonates (**10d**, **h, l, p**)

The Cu_2_I_2_ (1.14 g, 3.00 mmol) was suspended into dry THF (5.0 mL). To the reaction mixture phenylmagnesium chloride (3.00 mmol in THF) was added dropwise and stirred at room temperature for 15 minutes. After this time mixture was cooled to 0 ⁰C in ice – water bath and a solution of (2,2-dialkyl(diaryl)-4-oxo-3,4-dihydro-2*H*-pyran-5-yl)phosphonates (**9a – d**) (1.0 mmol) in dry THF (5 mL) was added dropwise. After 5 minutes ice – water was removed and reaction mixture was stirred for 3 hours at room temperature. After 3 hours saturated solution of ammonium chloride (15 mL) was added. The water layer was washed with DCM (3 x15 mL). Combined organic extracts were washed with brine (20 mL) and dried over MgSO_4_. The solvents were evaporated under reduced pressure and the resulting crude product was purified by column chromatography (eluent DCM : Acetone 20:1).

Diethyl (6,6-dimethyl-4-oxo-2-phenyltetrahydro-2H-pyran-3-yl)phosphonate (**10d**) (234.8 mg, 69%) Colorless oil. ^31^P NMR (101 MHz, Chloroform-*d*) δ 17.84 (trans), 17.96 (cis), 24.09 (enol). ^1^H NMR (700 MHz, Chloroform-*d*) δ 0.71 (t, *J* = 7.0 Hz, 3H, enol), 0.92 (t, *J* = 7.1 Hz, 3H, trans), 1.13 (t, *J* = 7.1 Hz, 3H, trans), 1.27 (s, 3H, trans), 1.29 (s, 3H, enol), 1.30 (s, 3H, enol), 1.32 (t, *J* = 7.0 Hz, 3H, enol), 1.38 (s, 3H, trans), 2.05 – 2.09 (m, 1H, enol), 2.45 (dd, *J* = 13.2, 1.1 Hz, 1H, trans), 2.51 (dd, *J* = 17.0, 3.1 Hz, 1H, enol), 2.88 (d, *J* = 13.2 Hz, 1H, trans), 3.10 – 3.17 (m, 1H, enol), 3.29 (ddd, *J* = 22.3, 10.0, 1.1 Hz, 1H, trans), 3.56 (m, 1H, enol), 3.60 – 3.73 (m, 2H, trans), 3.81 – 3.93 (m, 2H, enol), 3.96 – 4.06 (m, 2H, trans), 5.02 (dt, *J* = 2.8, 1.6 Hz, 1H, enol), 5.13 (dd, *J* = 37.3, 3.8 Hz, 1H, cis), 5.26 (dd, *J* = 10.0, 5.8 Hz, 1H, trans), 7.17 – 7.47 (m, 5H trans + 5H enol), 11.26 (s, 1H, enol). ^13^C NMR (176 MHz, Chloroform-*d*) δ 15.27 (d, *J* = 6,7 Hz, enol), 15.71 (d, *J* = 6.8 Hz, trans), 16.06 (d, *J* = 6.0 Hz, trans), 16.24 (d, *J* = 6.4 Hz, enol), 23.00 (enol), 26.00 (trans), 30.13 (trans), 30.19 (d, *J* = 1.6 Hz, enol), 40.11 (d, *J* = 11.7 Hz, enol), 51.57 (trans), 58.16 (d, *J* = 128.6 Hz, trans), 61.17 (d, *J* = 4.7 Hz, enol), 61.67 (d, *J* = 6.9 Hz, trans), 61.71 (d, *J* = 4.5 Hz, enol), 62.92 (d, *J* = 6.6 Hz, trans), 71.27 (enol), 72.57 (d, *J* = 14.0 Hz, enol), 74.41 (d, *J* = 2.4 Hz, trans), 75.63 (trans), 91.09 (d, *J* = 179.2 Hz, enol), 127.63 (trans, 2$\times$C), 127.88 (trans, 2$\times$C), 128.20 (enol, 2$\times$C), 128.30 (enol, 3$\times$C), 128.42 (trans), 139.78 (trans), 141.52 (enol), 167.47 (d, *J* = 4.1 Hz, enol), 202.51 (d, *J* = 6.0 Hz, trans). ESI-MS [M+Na]^+^ = 363.1. Anal. Calcd for C_17_H_25_O_5_P: C, 59.99; H, 7.40. Found: C, 59.90; H, 7.39.

Due to small amount of the cis form, the signals of cis form in the ^1^H and ^13^C NMR spectra have not been assigned.

Diethyl (4-oxo-2-phenyl-1-oxaspiro[5.5]undecan-3-yl)phosphonate (**10h**) (266.3 mg, 70%) ^31^P NMR (101 MHz, Chloroform-*d*) δ 18.14 (trans), 18.23 (cis), 24.29 (enol). ^1^H NMR (700 MHz, Chloroform-*d*) δ 0.69 (t, *J* = 7.1 Hz, 3H, enol), 0.86 – 0.93 (m, 3H trans + 3H cis), 1.07 – 1.14 (m, 3H trans + 3H cis), 1.17 – 2.02 (m, 10H trans + 10H cis + 13H enol), 2.14 (dd, *J* = 17.2, 1.2 Hz, 1H, enol), 2.31 – 2.37 (m, 1H cis + 1H enol), 2.44 (dd, *J* = 13.4, 1.2 Hz, 1H, trans), 2.78 (d, *J* = 13.4 Hz, 1H, trans), 2.93 (d, *J* = 13.3 Hz, 1H, cis), 3.06 – 3.14 (m, 1H, enol), 3.22 – 3.33 (m, 1H trans + 1H cis), 3.48 – 4.12 (m, 4H trans + 4H cis + 3H enol), 4.95 (dt, *J* = 2.9, 1.6 Hz, 1H, enol), 5.06 (dd, *J* = 37.3, 3.7 Hz, 1H, cis), 5.22 (dd, *J* = 10.0, 6.1 Hz, 1H, trans), 7.16 – 7.47 (m, 5H trans + 5H cis + 5H enol), 11.22 (s, 1H, enol). ^13^C NMR (176 MHz, Chloroform-*d*) δ 15.41 (d, *J* = 7.7 Hz, enol), 15.85 (d, *J* = 6.7 Hz, trans), 16.02 (d, *J* = 6.3 Hz, cis), 16.15 (d, *J* = 6.7 Hz, cis), 16.19 (d, *J* = 6.7 Hz, trans), 16.37 (d, *J* = 6.3 Hz, enol), 21.30 (cis), 21.74 (enol), 21.78 (trans), 21.88 (trans), 21.89 (cis), 22.25 (enol), 25.29 (trans), 25.41 (cis), 25.91 (enol), 31.52 (enol), 31.54 (cis), 34.45 (trans), 38.81 (trans), 39.19 (enol), 39.39 (cis), 50.71 (trans), 51.80 (cis), 58.55 (d, *J* = 128.3 Hz, trans), 59.07 (d, *J* = 129.6 Hz, cis), 61.27 (d, *J* = 4.5 Hz, enol), 61.79 (d, *J* = 7.0 Hz, trans), 61.80 (d, *J* = 4.5 Hz, enol), 61.97 (d, *J* = 6.9 Hz, cis), 62.06 (d, *J* = 6.9 Hz, cis), 63.01 (d, *J* = 6.4 Hz, trans), 71.27 (d, *J* = 7.3 Hz, cis), 71.63 (d, *J* = 13.6 Hz, enol), 72.45 (enol), 73.50 (d, *J* = 2.2 Hz, trans), 76.75 (trans), 76.82 (cis), 91.28 (d, *J* = 179.0 Hz, enol), 125.95 (cis, 2$\times$C), 127.51 (cis), 127.73 (trans, 2$\times$C), 127.89 (cis, 2$\times$C), 127.92 (enol, 2$\times$C), 128.04 (enol), 128.30 (trans, 2$\times$C), 128.36 (enol, 2$\times$C), 128.38 (trans), 138.85 (d, *J* = 5.1 Hz, cis), 140.27 (trans), 141.92 (enol), 167.57 (d, *J* = 4.4 Hz, enol), 202.85 (cis), 202.92 (d, *J* = 5.9 Hz, trans). ESI-MS [M+Na]^+^ = 403.1. Anal. Calcd for C_20_H_29_O_5_P: C, 63.15; H, 7.68. Found: C, 63.25; H, 7.67.

Due to small amount of the enol form, the signals of enol form in the ^1^H and ^13^C NMR spectra have not been assigned.

Diethyl (4-oxo-2,6,6-triphenyltetrahydro-2*H*-pyran-3-yl)phosphonate (**10l**) (255.5 mg, 55%) White solid. mp. 150 - 151 °C. White solid. ^31^P NMR (101 MHz, Chloroform-*d*) δ 17.71 , 18.07 , 23.98. ^1^H NMR (700 MHz, Chloroform-*d*) δ 0.75 (t, *J* = 7.0 Hz, 3H, enol), 0.95 (t, *J* = 7.1 Hz, 3H, trans), 0.99 (t, *J* = 7.0 Hz, 3H, enol), 1.14 (t, *J* = 7.0 Hz, 3H, trans), 2.97 – 3.03 (m, 1H, enol), 3.05 (ddd, *J* = 17.4, 4.4, 2.7 Hz, 1H, enol), 3.11 – 3.19 (m, 1H, enol), 3.23 (d, *J* = 13.9 Hz, 1H, trans), 3.36 (dd, *J* = 17.3, 1.5 Hz, 1H, enol), 3.53 – 3.64 (m, 2H enol + 3H trans), 3.69 – 3.79 (m, 2H, trans), 3.85 (dd, *J* = 14.0, 1.4 Hz, 1H, trans), 4.80 (d, *J* = 2.6 Hz, 1H, enol), 4.99 (dd, *J* = 37.5, 3.7 Hz, 1H, cis), 5.14 (dd, *J* = 10.4, 4.8 Hz, 1H, trans), 7.17 – 7.63 (m, 15H enol + 15H trans + 15H cis), 11.51 (d, *J* = 1.5 Hz, 1H, enol). ^13^C NMR (176 MHz, Chloroform-*d*) δ 15.42 (d, *J* = 8.0 Hz, enol), 15.84 (d, *J* = 6.7 Hz, trans), 16.04 (d, *J* = 7.4 Hz, enol), 16.17 (d, *J* = 6.3 Hz, trans), 29.75 (trans), 39.62 (d, *J* = 12.0 Hz, enol), 57.88 (d, *J* = 132.6 Hz, trans), 58.93 (d, *J* = 128.3 Hz, cis), 61.32 (d, *J* = 4.3 Hz, enol), 61.38 (d, *J* = 3.5 Hz, enol), 61.81 (d, *J* = 6.8 Hz, trans), 62.85 (d, *J* = 6.3 Hz, trans), 73.68 (d, *J* = 14.7 Hz, enol), 75.26 (d, *J* = 2.6 Hz, trans), 79.60 (enol), 82.77 (trans), 91.66 (d, *J* = 174.9 Hz, enol), 125.25 (enol, 2$\times$C), 126.96 (enol), 127.39 (enol, 2$\times$C), 127.59 (enol), 128.14 (enol), 128.16 (enol, 2$\times$C), 128.18 (enol, 2$\times$C), 128.55 (enol, 2$\times$C), 128.62 (enol, 2$\times$C), 139.67 (trans), 141.60 (enol), 141.69 (enol), 143.16 (trans), 146.44 (trans), 146.72 (enol), 167.85 (d, *J* = 4.1 Hz, enol), 201.30 (d, *J* = 6.0 Hz, trans). ESI-MS [M+Na]^+^ = 487.0. Anal. Calcd for C_27_H_29_O_5_P: C, 69.82; H, 6.29. Found: C, 69.95; H, 6.28

Due to small amount of the trans and cis forms, the signals of trans and cis forms in the ^1^H and ^13^C NMR spectra have not been assigned.

Diethyl (4'-oxo-6'-phenyl-3',4',5',6'-tetrahydrospiro[fluorene-9,2'-pyran]-5'-yl)phosphonate (**10p**) (346.9 mg, 75%) Yellow oil. ^31^P NMR (101 MHz, Chloroform-*d*) δ 17.68 (trans), 18.25 (cis), 22.87 (enol). ^1^H NMR (700 MHz, Chloroform-*d*) δ 0.73 (t, *J* = 7.0 Hz, 3H, enol), 0.85 (t, *J* = 7.1 Hz, 3H, cis), 0.94 (t, *J* = 7.1 Hz, 3H, trans), 1.15 (t, *J* = 7.0 Hz, 3H, trans), 1.19 (t, *J* = 7.1 Hz, 3H, cis), 1.35 (t, *J* = 7.1 Hz, 3H, enol), 2.27 (dd, *J* = 17.4, 1.2 Hz, 1H, enol), 2.40 – 2.51 (m, 1H, cis), 2.88 (dd, *J* = 14.0, 1.0 Hz, 1H, trans), 3.09 – 3.16 (m, 1H, enol), 3.17 – 3.24 (m, 1H, cis), 3.31 (ddd, *J* = 17.4, 4.3, 2.4 Hz, 1H, enol), 3.44 (d, *J* = 13.9 Hz, 1H, trans), 3.59 (ddd, *J* = 20.9, 3.8, 1.9 Hz, 1H, cis), 3.62 – 3.79 (m, 3H trans + 1H cis + 1H enol), 3.89 (d, *J* = 13.7 Hz, 1H, cis), 3.91 – 4.20 (m, 2H trans + 2H cis + 2H enol), 5.38 (d, *J* = 2.0 Hz, 1H, enol), 5.69 (dd, *J* = 37.1, 3.6 Hz, 1H, cis), 5.86 (dd, *J* = 10.1, 6.1 Hz, 1H, trans), 6.93 – 8.09 (m, 13H trans + 13H cis + 13H enol), 11.42 (s, 1H, enol). ^13^C NMR (176 MHz, Chloroform-*d*) δ 15.46 (d, *J* = 7.7 Hz, enol), 15.88 (d, *J* = 6.6 Hz, trans), 16.02 (d, *J* = 6.4 Hz, cis), 16.27 (d, *J* = 6.2 Hz, cis), 16.28 (d, *J* = 6.3 Hz, trans), 16.47 (d, *J* = 6.4 Hz, enol), 36.69 (d, *J* = 12.0 Hz, enol), 47.29 (trans), 47.81 (cis), 58.84 (d, *J* = 128.3 Hz, trans), 59.33 (d, *J* = 129.5 Hz, cis), 61.68 (d, *J* = 4.8 Hz, enol), 62.08 (d, *J* = 7.0 Hz, trans), 62.18 (d, *J* = 6.8 Hz, enol), 62.26 (d, *J* = 6.8 Hz, cis), 62.44 (d, *J* = 7.1 Hz, cis), 63.41 (d, *J* = 6.4 Hz, trans), 74.88 (d, *J* = 7.1 Hz, cis), 75.07 (d, *J* = 13.8 Hz, enol), 76.40 (d, *J* = 2.3 Hz, trans), 81.39 (enol), 84.22 (trans), 84.71 (cis), 93.15 (d, *J* = 181.3 Hz, enol), 119.75 (cis), 119.84 (enol), 119.96 (trans), 120.48 (trans), 120.53 (enol), 120.80 (cis), 123.95 (trans), 124.08 (enol), 124.42 (trans), 124.60 (enol), 124.98 (cis), 125.05 (cis), 126.10 (cis, 2$\times$C), 127.35 (cis), 127.69 (enol), 127.72 (cis, 2$\times$C), 127.91 (trans, 2$\times$C), 128.07 (enol, 2$\times$C), 128.10 (enol), 128.17 (enol), 128.39 (trans), 128.42 (trans 3$\times$C + enol), 128.72 (trans), 129.45 (enol), 129.53 (enol), 129.60 (cis), 129.63 (trans), 129.71 (trans), 129.76 (cis), 138.83 (cis), 138.92 (trans), 139.48 (enol), 139.55 (trans), 140.04 (enol), 140.53 (trans), 140.58 (cis), 141.22 (enol), 144.53 (cis), 144.86 (enol), 145.68 (trans), 146.50 (cis), 146.73 (enol), 146.97 (trans), 167.02 (d, *J* = 4.4 Hz, enol), 201.80 (d, *J* = 6.2 Hz, trans), 202.14 (enol). ESI-MS [M+Na]^+^ = 485.0. Anal. Calcd for C_27_H_27_O_5_P: C, 70.12; H, 5.88. Found: C, 69.98; H, 5.89.

Due to small amount of the cis form, the signals of cis form in the ^1^H and ^13^C NMR spectra have not been assigned.

3. Copies of ^31^P, ^1^H and ^13^C NMR Spectra.

Diethyl (4-hydroxy-4-methyl-2-oxopentyl)phosphonate (**7a**)

Diethyl (3-(1-hydroxycyclohexyl)-2-oxopropyl)phosphonate (**7b**)

Diethyl (4-hydroxy-2-oxo-4,4-diphenylbutyl)phosphonate (**7c**)

Diethyl (3-(9-hydroxy-9*H*-fluoren-9-yl)-2-oxopropyl)phosphonate (**7d**)

Diethyl (2,2-dimethyl-4-oxo-3,4-dihydro-2*H*-pyran-5-yl)phosphonate (**9a**)

Diethyl (4-oxo-1-oxaspiro[5.5]undec-2-en-3-yl)phosphonate (**9b**)

Diethyl (4-oxo-2,2-diphenyl-3,4-dihydro-2*H*-pyran-5-yl)phosphonate (**9c**)

Diethyl (4'-oxo-3',4'-dihydrospiro[fluorene-9,2'-pyran]-5'-yl)phosphonate (**9d**)

Diethyl (2-ethyl-6,6-dimethyl-4-oxotetrahydro-2*H*-pyran-3-yl)phosphonate (**10a**)

Diethyl (2-butyl-6,6-dimethyl-4-oxotetrahydro-2*H*-pyran-3-yl)phosphonate (**10b**)

Diethyl (2-isopropyl-6,6-dimethyl-4-oxotetrahydro-2*H*-pyran-3-yl)phosphonate (**10c**)

Diethyl (6,6-dimethyl-4-oxo-2-phenyltetrahydro-2*H*-pyran-3-yl)phosphonate (**10d**)

Diethyl (2-ethyl-4-oxo-1-oxaspiro[5.5]undecan-3-yl)phosphonate (**10e**)

Diethyl (2-butyl-4-oxo-1-oxaspiro[5.5]undecan-3-yl)phosphonate (**10f**)

Diethyl (2-isopropyl-4-oxo-1-oxaspiro[5.5]undecan-3-yl)phosphonate (**10g**)

Diethyl (4-oxo-2-phenyl-1-oxaspiro[5.5]undecan-3-yl)phosphonate (**10h**)

Diethyl (2-ethyl-4-oxo-6,6-diphenyltetrahydro-2*H*-pyran-3-yl)phosphonate (**10i**)

Diethyl (2-butyl-4-oxo-6,6-diphenyltetrahydro-2*H*-pyran-3-yl)phosphonate (**10j**)

Diethyl (2-isopropyl-4-oxo-6,6-diphenyltetrahydro-2*H*-pyran-3-yl)phosphonate (**10k**)

Diethyl (4-oxo-2,6,6-triphenyltetrahydro-2*H*-pyran-3-yl)phosphonate (**10l**)

Diethyl (6'-ethyl-4'-oxo-3',4',5',6'-tetrahydrospiro[fluorene-9,2'-pyran]-5'-yl)phosphonate (**10m**)

Diethyl (6'-butyl-4'-oxo-3',4',5',6'-tetrahydrospiro[fluorene-9,2'-pyran]-5'-yl)phosphonate (**10n**)

Diethyl (6'-isopropyl-4'-oxo-3',4',5',6'-tetrahydrospiro[fluorene-9,2'-pyran]-5'-yl)phosphonate (**10o**)

Diethyl (4'-oxo-6'-phenyl-3',4',5',6'-tetrahydrospiro[fluorene-9,2'-pyran]-5'-yl)phosphonate (**10p**)

6-Ethyl-2,2-dimethyl-5-methylidenetetrahydro-4*H*-pyran-4-one (**11a**)

6-Butyl-2,2-dimethyl-5-methylidenetetrahydro-4*H*-pyran-4-one (**11b**)


6-Isopropyl-2,2-dimethyl-5-methylidenetetrahydro-4*H*-pyran-4-one (**11c**)


2,2-Dimethyl-5-methylidene-6-phenyltetrahydro-4*H*-pyran-4-one (**11d**)


2-Ethyl-3-methylidene-1-oxaspiro[5.5]undecan-4-one (**11e**)


2-Butyl-3-methylidene-1-oxaspiro[5.5]undecan-4-one (**11f**)


2-Isopropyl-3-methylidene-1-oxaspiro[5.5]undecan-4-one (**11g**)


3-Methylidene-2-phenyl-1-oxaspiro[5.5]undecan-4-one (**11h**)


6-Ethyl-5-methylidene-2,2-diphenyltetrahydro-4*H*-pyran-4-one (**11i**)

6-Butyl-5-methylidene-2,2-diphenyltetrahydro-4*H*-pyran-4-one (**11j**)


6-Isopropyl-5-methylidene-2,2-diphenyltetrahydro-4*H*-pyran-4-one (**11k**)


5-Methylidene-2,2,6-triphenyltetrahydro-4*H*-pyran-4-one (**11l**)

6'-Ethyl-5'-methylidene-5',6'-dihydrospiro[fluorene-9,2'-pyran]-4'(3'*H*)-one (**11m**)


6'-Butyl-5'-methylidene-5',6'-dihydrospiro[fluorene-9,2'-pyran]-4'(3'*H*)-one (**11n**)


6'-Isopropyl-5'-methylidene-5',6'-dihydrospiro[fluorene-9,2'-pyran]-4'(3'*H*)-one (**11o**)

5'-Methylidene-6'-phenyl-5',6'-dihydrospiro[fluorene-9,2'-pyran]-4'(3'*H*)-one (**11p**)
